# Case report: Malignant vasovagal reflex syndrome during percutaneous transcatheter closure of patent foramen ovale

**DOI:** 10.3389/fcvm.2023.1150011

**Published:** 2023-07-04

**Authors:** Zhiyang Li, Qian Dong, Lun Huang, Qiutang Zeng, Kunwu Yu

**Affiliations:** Department of Cardiology, Union Hospital, Tongji Medical College, Huazhong University of Science and Technology, Wuhan, China

**Keywords:** vasovagal reflex syndrome, percutaneous transcatheter closure of patent foramen ovale, cardiac arrest, ventricular tachycardia, malignant arrhythmia

## Abstract

Malignant vasovagal reflex syndrome can be induced by pulling of cardiac tissue during percutaneous transcatheter closure of patent foramen ovale. In this case, a patient presented with a malignant vasovagal reflex syndrome characterized by decreased heart rate, cardiac arrest, and ventricular tachycardia. Therefore, it's particularly important to observe patients' heart rate and timely deal with vasovagal reflex syndrome during the operation.

## Introduction

With the rapid development of cardiac catheterization, interventional cardiac therapy has been widely applied in the treatment of a variety of heart diseases. But at the same time, there are plenty of related complications, vasovagal reflex syndrome (VVRS) is one of them. We report that a middle-aged female patient diagnosed with patent foramen ovale (PFO) developed cardiac arrest and ventricular tachycardia during percutaneous transcatheter closure of PFO.

## Case presentation

**Figure 1:**
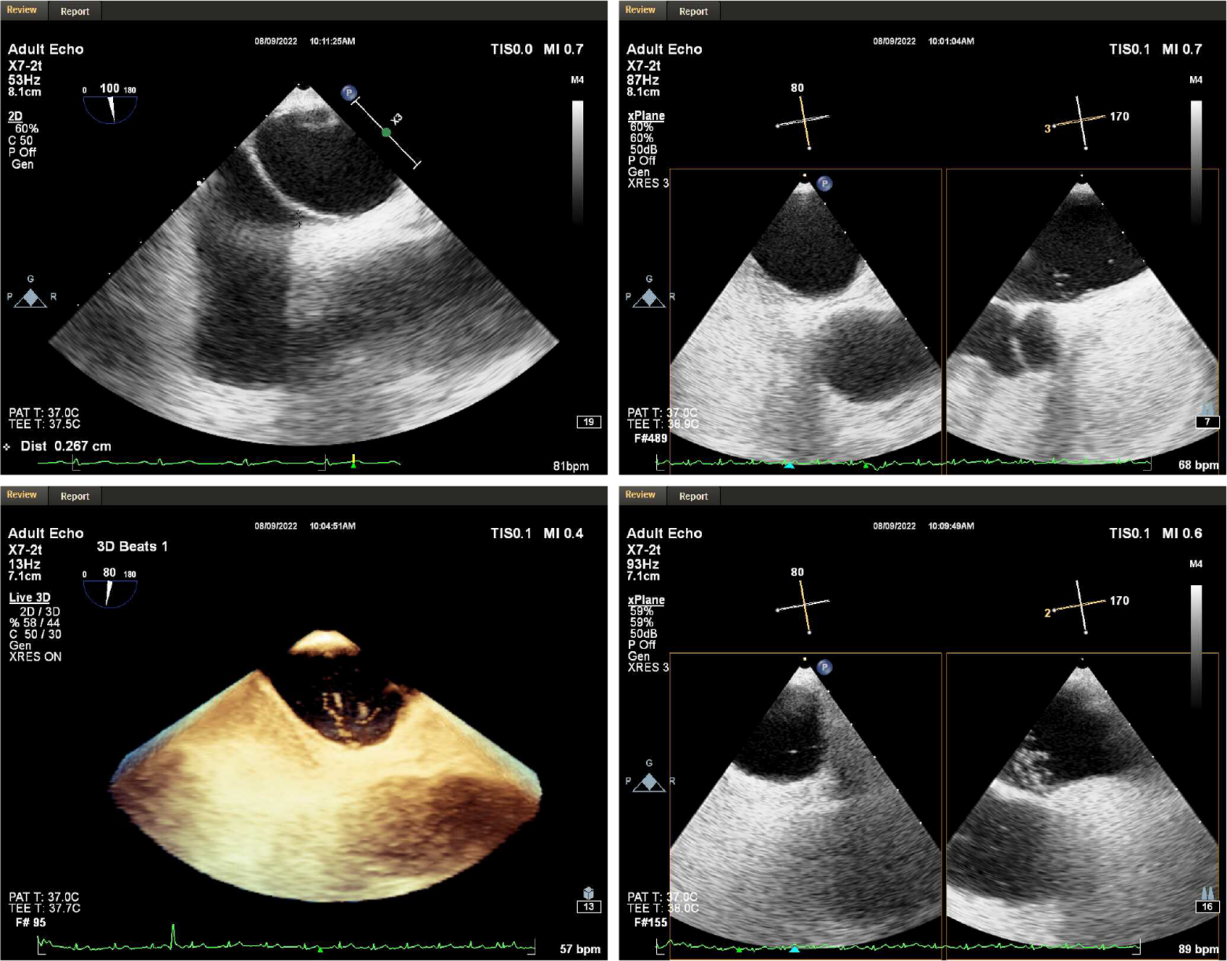
Transesophageal echocardiography. A PFO with a length of 9.7 mm, a diameter of 2.7 mm in the right atrium and 1.1 mm in the left atrium was observed in the middle of the patient's atrial septum. At rest, a moderate amount of shunt was seen in the left atrium, and a large number of shunt was seen immediately after increasing abdominal pressure.

**Figure 2:**
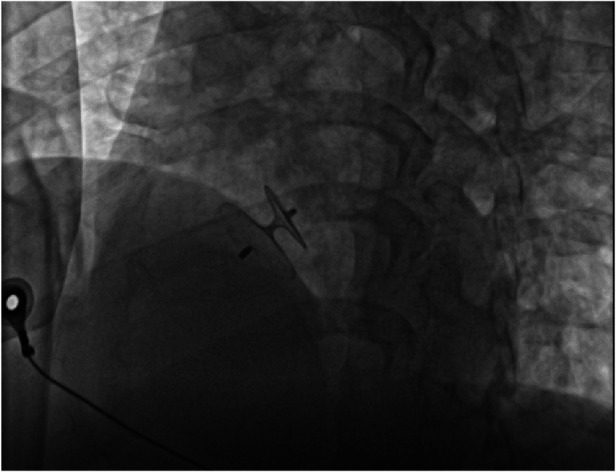
Intraoperative placement of foramen ovale occlusive device. The size of occluder is 18 mm × 25 mm.

**Figure 3:**
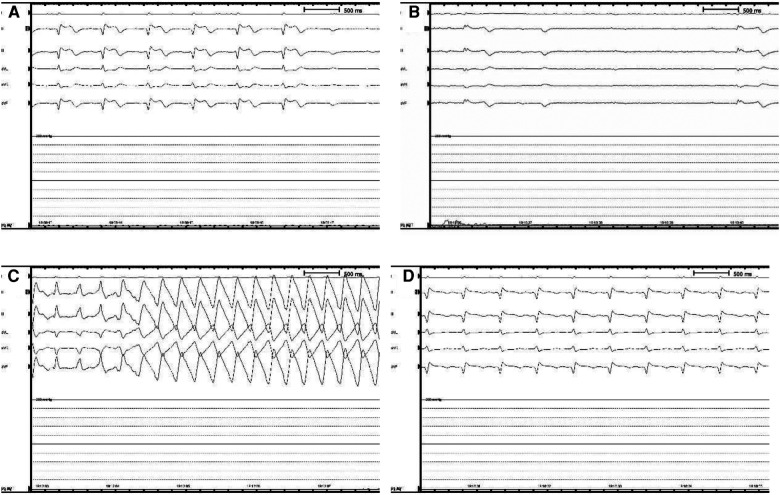
Intraoperative holter monitoring. (**A**) The patient began to show a slow heart rate and ST segment elevation in ll, lll, and avF leads, presenting as a junctional escape rhythm. (**B**) The patient went into cardiac arrest that lasted 3.8 s. (**C**) After heartbeat recovery, the patient developed ventricular tachycardia, with a maximum ventricular rate of 240 beats per minute. (**D**) After rapid chest compressions, electrical defibrillation, intravenous epinephrine (5 mg), sputum aspiration, oxygen inhalation, and fluid replenishment, the patient returned to sinus rhythm.

A 51-year-old woman presented with intermittent tightness in her chest and headaches for 10 years, aggravated 2 months. Transesophageal echocardiography showed normal biventricular function (left ventricular ejection fraction of 65%) and a bidirectional shunt upon saline flush test, indicative of a PFO (severe right-to-left shunt via a PFO 2.7 mm in diameter and 9.7 mm tunnel length during Valsalva maneuver) (Figure 1). No obvious arrhythmia was observed by Holter monitor, and headache caused by craniocerebral lesions was excluded by craniocerebral MRI. Taking into account the patient's age and history of hypertension, we calculated the paradoxical embolism risk score, which concluded that the patient was at higher risk of stroke, combined with the patient's chronic suffering from unexplained migraine. Therefore, percutaneous transcatheter closure of PFO is appropriate. For the selection of the size of the occluder, we usually choose an device with a length of about twice the length of the PFO and a diameter slightly larger than the PFO diameter for treatment. We chose to use an 18 mm × 25 mm occluder for this operation (Figure 2). The patient had sinus rhythm before operation, which is 65 beats per minute. During the distraction test of the foramen ovale occluder, the patient developed a sudden and severe VVRS with a significant decrease in heart rate and blood pressure. There was no improvement after rapid intravenous administration of 1 mg atropine. The patient then went into cardiac arrest. After rapid chest compressions, intravenous administration of epinephrine (5 mg), sputum aspiration, oxygen inhalation, and fluid replenishment, the patient's heart resumed beating. The duration of cardiac arrest was 3.8 s, but ventricular tachycardia occurred repeatedly after the recovery of heart beat, with a maximum ventricular rate of 240 beats per minute. After five cycles of bidirectional electrical defibrillation at 150 J, sinus rhythm was successfully restored, vital signs were stable, and the percutaneous closure of the PFO was successfully performed (Figure 3). Post-operative echocardiography showed that the shape and position of the occluder were normal and there was no significant residual shunt. Three months after discharge, the patient underwent echocardiography again. The left ventricular ejection fraction was 73%, the internal diameter of the right atrium changed from the initial 3.2 cm to 3.5 cm, and no residual shunt signal was found in the occlusion area. At the same time, there was no recurrence of malignant arrhythmia events.

## Discussion

VVRS is a common adverse reaction in interventional cardiovascular therapy. Under physiological conditions, cardiomyocytes and conduction system of the heart are innervated by efferent fibers of the posterior vagal nerve ganglia (1). Increase in efferent activity of the vagal nerve can be generally seen in following situations: (1) activating the central axis and directly stimulating the vagal nerve to increase neuronal firing and Ach releasing; (2) the administration of cholinergic drugs or cholinesterase inhibitors to increase acetylcholine (ACH) levels indirectly; (3) improving the availability of ACH by activating ACH related receptors and downstream pathways (2). In addition, arrhythmias caused by increased vagal reactivity are also not rare in heart-related diseases. In patients with Brugada syndrome, an increased cholinergic tone may exert its arrhythmogenic effect by increasing dispersion of transmural repolarization during autonomic dysfunction (3), which also suggests a high incidence of cardiac arrest at night. In patients with hypertrophic cardiomyopathy, the presence of left ventricular outflow tract obstruction reduces diastolic filling and cardiac output, which is also one of the causes of malignant arrhythmias (4). Besides, age and gender are also important factors in the occurrence of VVRS (5).

The malignant VVRS in this case may be due to the excitation of mechanical sensors in heart during the percutaneous transcatheter closure of PFO, further transmit information to the nucleus of tractus solitarius, causing vagal nerve reflexed, excited cholinergic nerve, nerve endings released acetylcholine on M receptor, resulting myocardial contractility decreased significantly, heart rate decreased, peripheral vascular dilation, blood pressure decreased (6). Under fluoroscopy, the amplitude of myocardial contraction was significantly reduced. Subsequent ventricular tachycardia, however, may have been triggered by the high levels of adrenaline used to restore the heart's electrical activity. ​The occurrence of VVRS during cardiac interventional therapy is relatively common, but this case is the first in recent years to report severe VVRS during operation that resulted in cardiac arrest.

**Figure 4:**
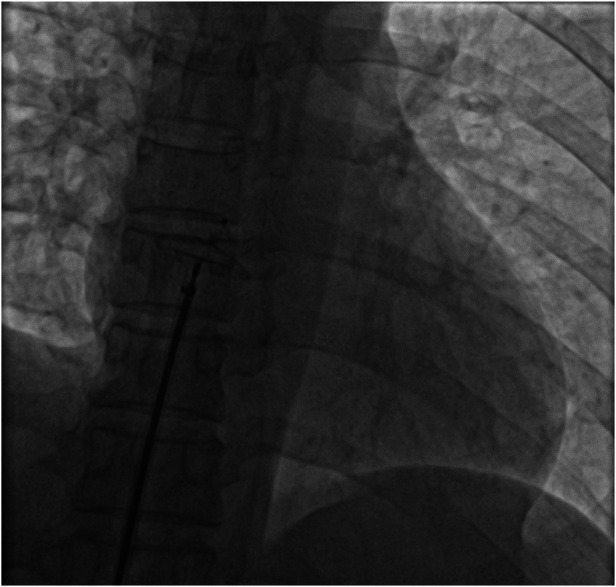
Pericardial tamponade was excluded by fluoroscopy during operation.

**Figure 5:**
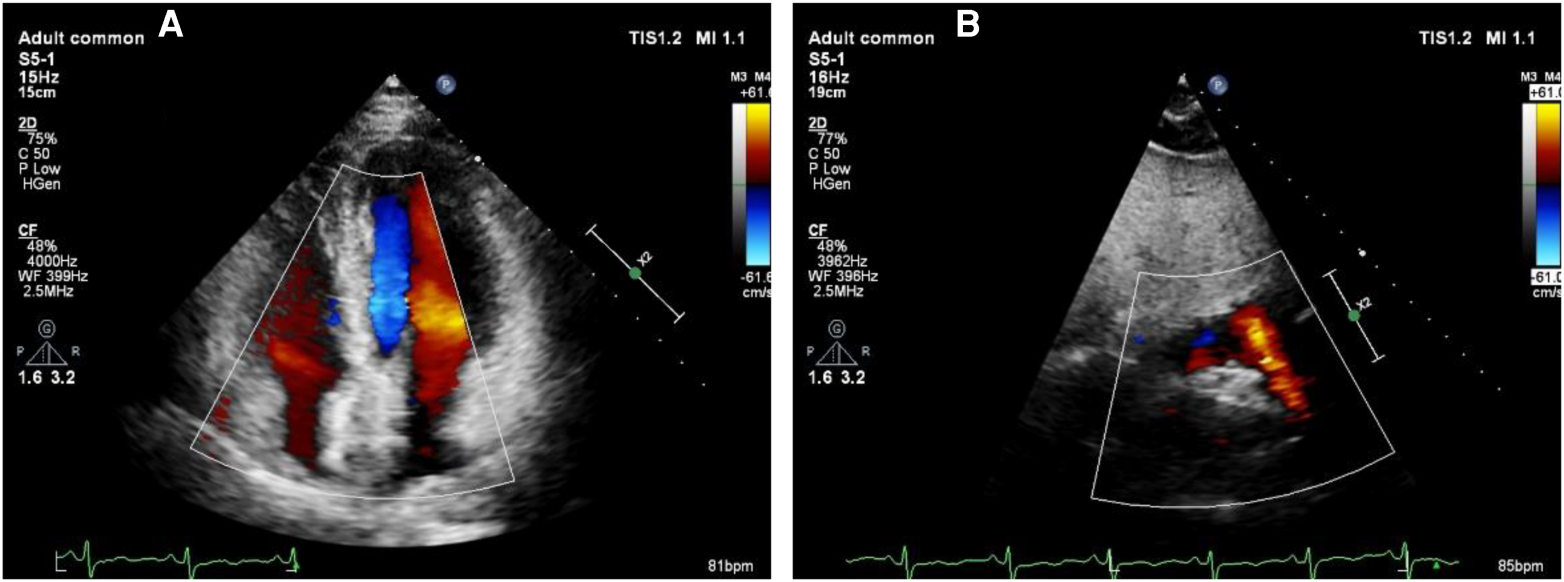
Echocardiography to exclude pericardial tamponade. (**A**) The left ventricular ejection fraction of 65% with no evidence of pericardial tamponade was found. (**B**) Strong echo of the occluder can be seen in atrial septum.

In percutaneous transcatheter closure of PFO, pericardial tamponade, as one of the serious complications of therapy, can also lead to decreased heart rate, decreased blood pressure and even cardiac arrest (7). The main causes of pericardial tamponade include: (1) the guide wire punctures the atrial wall; (2) when the infusion tube is placed in the left upper pulmonary vein, if the sheath tube is poorly fixed, it may cause atrial rupture when the occluder is pushed. At the same time, heparinization makes pericardial tamponade more likely after atrial rupture. Therefore, when a patient's heart rate drops during procedure, in addition to considering the VVRS, more attention should be paid to the occurrence of pericardial tamponade. And the occurrence of pericardial tamponade can be identified by cardiac ultrasound or intraoperative fluoroscopy. In this case, we performed intraoperative fluoroscopy (Figure 4) and echocardiography (Figure 5) to rule out pericardial tamponade. It is important to note that delayed pericardial effusion can also be fatal if the occlusive device is improperly positioned, such as blocking the coronary sinus (8).

The improvement in the management of this case is that we can prevent further heart rate drops and even cardiac arrest by administering atropine earlier and more promptly when heart rate slows down. This also reminds us to keep a close eye on heart rate changes as the catheter enters the left atrium and the occluder is opened, in addition to being gentle during the procedure. Once the heart rate slows significantly, atropine should be injected immediately, and the possibility of pericardial tamponade should be ruled out. However, for patients with slow basal heart rate, even preoperative medication to raise heart rate may be necessary. A patient with a history of arrhythmia is at a higher risk of developing malignant arrhythmias during or after operation (9, 10). There also have been reports that the style and size of implanted devices are associated with arrhythmias during and even after operation. The incidence of arrhythmia events will be smaller with smaller implanted devices (11, 12).

VVRS is not uncommon during cardiac interventional procedures, but this does not mean that it should be neglected. VVRS has also been reported after venipuncture or even vaccination (13, 14). This indicates that VVRS is not confined to the heart but is present throughout the circulation system. When VVRS occurs, it may cause serious injury or even death if not treated promptly.

## Conclusion

As illustrated in this case, the patient's heart rate should be closely monitored during the percutaneous cardiac catheterization intervention. Atropine should be administered in advance if necessary to prevent the occurrence of VVRS, and vigilance should be maintained for the occurrence of pericardial tamponade.

## Data Availability

The original contributions presented in the study are included in the article, further inquiries can be directed to the corresponding authors.

## References

[1] Capilupi MJ Kerath SM Becker LB. Vagus nerve stimulation and the cardiovascular system. Cold Spring Harb Perspect Med. (2020) 10:1–3. 10.1101/cshperspect.a034173 PMC6996447 31109966

[2] Liu L Zhao M Yu X Zang W. Pharmacological modulation of vagal nerve activity in cardiovascular diseases. Neurosci Bull. (2019) 35:156–66. 10.1007/s12264-018-0286-7 30218283 PMC6357265

[3] Mascia G Bona RD Ameri P Canepa M Porto I Parati G Brugada syndrome and syncope: a practical approach for diagnosis and treatment. Europace. (2021) 23:996–1002. 10.1093/europace/euaa370 33367713

[4] Williams L Frenneaux M. Syncope in hypertrophic cardiomyopathy: mechanisms and consequences for treatment. Europace. (2007) 9:817–22. 10.1093/europace/eum093 17522079

[5] Olde Nordkamp LR Ruwald MH Goldenberg I Wieling W McNitt S Polonsky B Syncope in genotype-negative long QT syndrome family members. Am J Cardiol. (2014) 114:1223–8. 10.1016/j.amjcard.2014.07.044 25173441

[6] Goldberger JJ Arora R Buckley U Shivkumar K. Autonomic nervous system dysfunction: JACC focus seminar. J Am Coll Cardiol. (2019) 73:1189–206. 10.1016/j.jacc.2018.12.064 30871703 PMC6958998

[7] Merkler AE Gialdini G Yaghi S Okin PM Iadecola C Navi BB Safety outcomes after percutaneous transcatheter closure of patent foramen Ovale. Stroke. (2017) 48:3073–7. 10.1161/STROKEAHA.117.018501 28939677 PMC5699514

[8] Shen QS Li DY Jin J Jing C. A case of delayed pericardial effusion after device closure of PFO: an unsuspected cause. Intensive Care Med. (2023) 49:237–8. 10.1007/s00134-022-06929-1 36418511

[9] Johnson JN Marquardt ML Ackerman MJ Asirvatham SJ Reeder GS Cabalka AK Electrocardiographic changes and arrhythmias following percutaneous atrial septal defect and patent foramen ovale device closure. Catheter Cardiovasc Interv. (2011) 78:254–61. 10.1002/ccd.23028 21563292

[10] Silversides CK Siu SC McLaughlin PR Haberer KL Webb GD Benson L Symptomatic atrial arrhythmias and transcatheter closure of atrial septal defects in adult patients. Heart. (2004) 90:1194–8. 10.1136/hrt.2003.022475 15367523 PMC1768500

[11] Komar M Przewlocki T Prochownik P Gancarczyk U Sobien B Libiszewska N 285Arrhythmias After transcatheter closure of persistent foramen ovale are related to the type and size of the implanted device. Eur Heart J. (2019) 40(Issue Supplement_1):1. 10.1093/eurheartj/ehz747.008930602013

[12] Scapinelli M Campisano M Bagni E. P33 supraventricular tachyarrhythmias and recurrent syncopes after percutaneous closure of PFO in a young patient with no history of thromboembolism. Importance of current guidelines. Eur Heart J Suppl. (2022) 24:1. 10.1093/eurheartj/suac012.031

[13] Mizumura N Kishimoto T Tanaka T Shimizu J Tabata T Eguchi Y. Vasovagal reaction and ischemic colitis following blood donation. Intern Med. (2020) 59:1515–7. 10.2169/internalmedicine.4219-19 32188808 PMC7364255

[14] Takase B Hayashi K Takei S Hisada T Masaki N Nagata M. Delayed vasovagal reaction with reflex syncope following COVID-19 vaccination. Intern Med. (2022) 61:2167–70. 10.2169/internalmedicine.9318-21 35569982 PMC9381344

